# Geraniol Suppresses Angiogenesis by Downregulating Vascular Endothelial Growth Factor (VEGF)/VEGFR-2 Signaling

**DOI:** 10.1371/journal.pone.0131946

**Published:** 2015-07-08

**Authors:** Christine Wittig, Claudia Scheuer, Julia Parakenings, Michael D. Menger, Matthias W. Laschke

**Affiliations:** Institute for Clinical & Experimental Surgery, University of Saarland, Homburg/Saar, Germany; Ottawa Hospital Research Institute, CANADA

## Abstract

Geraniol exerts several direct pharmacological effects on tumor cells and, thus, has been suggested as a promising anti-cancer compound. Because vascularization is a major precondition for tumor growth, we analyzed in this study the anti-angiogenic action of geraniol. In vitro, geraniol reduced the migratory activity of endothelial-like eEND2 cells. Western blot analyses further revealed that geraniol downregulates proliferating cell nuclear antigen (PCNA) and upregulates cleaved caspase-3 (Casp-3) expression in eEND2 cells. Moreover, geraniol blocked vascular endothelial growth factor (VEGF)/VEGFR-2 signal transduction, resulting in a suppression of downstream AKT and ERK signaling pathways. In addition, geraniol significantly reduced vascular sprout formation in a rat aortic ring assay. In vivo, geraniol inhibited the vascularization of CT26 tumors in dorsal skinfold chambers of BALB/c mice, which was associated with a smaller tumor size when compared to vehicle-treated controls. Immunohistochemical analyses confirmed a decreased number of Ki67-positive cells and CD31-positive microvessels with reduced VEGFR-2 expression within geraniol-treated tumors. Taken together, these findings indicate that geraniol targets multiple angiogenic mechanisms and, therefore, is an attractive candidate for the anti-angiogenic treatment of tumors.

## Introduction

Angiogenesis, i.e. the formation of new blood vessels from pre-existing ones, is a key process in tumor pathogenesis. In fact, growing tumors are crucially dependent on an adequate blood supply, providing them with oxygen and essential nutrients [1]. Moreover, a newly developing tumor microvasculature enables metastatically-competent cells to depart from the primary tumor site and colonize initially unaffected organs [2]. Based on these considerations, anti-angiogenic therapy has rapidly evolved within the last three decades and is now an integral component of current standard treatment regimens in clinical oncology [3, 4]. Accordingly, there is also a continuous search for novel compounds, which suppress angiogenesis and exhibit a tolerable side effect profile.

The acyclic monoterpene geraniol naturally occurs in small quantities in geranium, lemon and other essential oils from medical plants and is the aromatical component in many cosmetic products. Beside its aromatic properties, geraniol also exhibits anti-oxidative [5, 6], anti-microbial [7, 8] and anti-inflammatory activity [9]. Moreover, it has been shown to suppress the growth of different tumor types by targeting cell cycle and apoptosis pathways [10–12]. For these reasons, the compound is currently discussed as a promising candidate for the development of novel chemopreventive or therapeutic approaches against cancer [13–16].

Recently, preventive application of geraniol has been reported to inhibit the expression of vascular endothelial growth factor (VEGF) in the buccal mucosa of hamsters in a model of 7,12-dimethylbenz(a)anthracene-induced buccal pouch carcinogenesis [17]. This preliminary finding indicates that geraniol may directly target the process of blood vessel formation. However, the effect of geraniol on angiogenesis is completely unknown so far. Therefore, we analyzed in this study the action of geraniol on viability, actin stress fiber formation, migration, and protein expression of murine endothelial-like eEND2 cells and on vascular sprout formation in a rat aortic ring assay. In addition, we generated spheroids of the murine colon carcinoma cell line CT26. These spheroids were then transplanted into the dorsal skinfold chamber of geraniol-treated and vehicle-treated BALB/c mice for the in vivo analysis of tumor vascularization and growth.

## Materials and Methods

### Cell culture

For the in vitro angiogenesis assays, we used murine endothelial-like eEND2 cells (kind gift of Henrik Thorlacius, 2005, Department of Surgery, Malmö Hospital, Lund University, Malmö, Sweden). The cells were cultured in Dulbecco’s modified Eagle’s medium (DMEM; PAA, Cölbe, Germany) supplemented with 10% fetal calf serum (FCS), 100U/mL penicillin and 0.1mg/mL streptomycin (PAA). In addition, we used human dermal microvascular endothelial cells (HDMEC; PromoCell, Heidelberg, Germany), which were cultured in EC-MV complete medium (PromoCell). For the in vivo tumor experiments, we used the CT26 cell line (ATCC CRL-2638; LGC Promochem GmbH, Wesel, Germany), which originates from a N-nitroso-N-methylurethane-induced undifferentiated colon carcinoma of the BALB/c mouse [18]. The cells were cultured in RPMI-1640 medium (PAA) supplemented with 10% FCS, 100U/mL penicillin and 0.1mg/mL streptomycin (PAA). All cell lines were cultured at 37°C in a humidified atmosphere of 5% CO_2_.

Geraniol with a purity of 99% was purchased from Sigma-Aldrich (Taufkirchen, Germany). A stock solution of geraniol (5M dissolved in dimethyl sulfoxide (DMSO)) was stored at -20°C. For the in vitro experiments, the stock solution was further diluted with the cell culture medium, resulting in geraniol concentrations of 50–400μM. Geraniol-treated and vehicle-treated cells were exposed to identical DMSO end volumes.

### Water-soluble tetrazolium (WST)-1 assay

The effect of geraniol on the viability of eEND2 cells and HDMEC was analyzed by means of a WST-1 assay (Roche diagnostics, Mannheim, Germany). For this purpose, 1 x 10^4^ eEND2 cells or HDMEC per well were seeded in 96-well plates and treated with vehicle (DMSO; control; n = 4), 1% Triton X-100 (Carl Roth GmbH, Karlsruhe, Germany) as cytotoxic control or different concentrations of geraniol (50, 100, 200, 400μM; n = 4 each). After 24h, 10μL of WST-1 reagent per 100μL medium was added to each well and after 30min incubation at 37°C the absorbance was measured at 450nm with 620nm as a reference using a microplate reader.

### Lactate dehydrogenase (LDH) release assay

Cytotoxic effects of geraniol on eEND2 cells and HDMEC were assessed by means of a LDH release assay using the Cytotoxicity Detection KitPLUS (Roche diagnostics). Briefly, 1 x 10^4^ eEND2 cells or HDMEC were seeded in 96-well plates and cultured with vehicle (DMSO; control; n = 4), 1% Triton X-100 as cytotoxic control or serial dilutions of geraniol (50, 100, 200, 400μM; n = 4 each). After a culture period of 24h, 100μL of reaction mix per 100μL medium was added to each well. The reaction was stopped after 10min at room temperature in the dark by 50μL stop solution. Subsequently, the absorbance was measured with a microplate reader at 492nm with 620nm as reference wavelength.

### Flow cytometry

The effect of geraniol on necrotic and apoptotic cell death of eEND2 cells and HDMEC was analyzed by means of flow cytometry. For this purpose, 1 x 10^5^ eEND2 cells or HDMEC per well were seeded in 6-well plates and treated with vehicle (DMSO; control; n = 4), 200 or 400μM geraniol (n = 4 each). After 24h, the cells were stained with the necrosis marker propidium iodide (PI; 1μg/mL; BD Biosciences, Heidelberg, Germany) and the apoptosis marker fluorescein isothiocyanat (FITC)-conjugated annexin V (1:150; BD Biosciences) and analyzed using a FACScan (BD Pharmingen, Heidelberg, Germany). Data were evaluated by the software package CellQuest (BD Pharmingen).

### Cytoskeleton staining

The effect of geraniol on the cytoskeleton organization of eEND2 cells was analyzed by phalloidin staining of filamentous actin. The cells were plated on sterile coverslips in a 24-well plate (1 x 10^5^ per well) and incubated in DMEM culture medium. After 24h, the cells were incubated in DMEM culture medium supplemented with 400μM geraniol or vehicle (DMSO) for another 24h. Then, the cells were fixed and stained with Alexa Fluor 568-conjugated phalloidin (Invitrogen, Darmstadt, Germany) and Hoechst 33342 (Sigma-Aldrich), as previously described in detail [18]. The cell bearing slides were quantitatively analyzed using an all in one Biozero BZ-8000 fluorescence microscope (Keyence, Neu-Isenburg, Germany). For this purpose, we assessed the fraction of cells with actin stress fibers (given as % of all analyzed cells) in 10 microscopic regions of interest (ROIs; size: 0.15mm²) at 20x magnification on 4 slides per group.

### Transwell migration assay

To assess the migratory activity of eEND2 cells, a 24-well chemotaxis chamber and polyethylene therephthalate (PET) filters with 8μm pore size (BD Biosciences) were used. eEND2 cells were exposed to 100, 200 or 400μM geraniol (n = 4 each) or vehicle (DMSO; control; n = 4) for 24h. For the analysis of cell migration, 500μL of a cell suspension, containing 1 x 10^5^ geraniol-treated or vehicle-treated cells in DMEM was added to each of the upper wells. The lower wells were filled with 750μL DMEM supplemented with 1% FCS. The chamber was then incubated for 6h at 37°C in a humidified atmosphere with 5% CO_2_. After the 6h incubation period, non-migrated cells were quantitatively removed from the upper surface of the filters. Migrated cells, which were adherent to the lower surface, were fixed with methanol and stained with Dade Diff-Quick (Dade Diagnostika GmbH, München, Germany). The number of these migrated cells was counted in 20 microscopic ROIs (size: 0.15mm²) at 20x magnification (Biozero BZ-8000; Keyence) and is given as cells/ROI.

### Western blot analysis

To determine geraniol effects on the expression of proliferating cell nuclear antigen (PCNA), cleaved caspase-3 (Casp-3), vascular endothelial growth factor receptor 2 (VEGFR-2), phosphorylated protein kinase B (pAKT), AKT, phosphorylated extracellular-signal regulated kinase (pERK) and ERK in eEND2 cells, the cells were cultured in medium supplemented with vehicle (DMSO; control), 200 or 400μM geraniol (n = 3 each). After 24h the cells were harvested with accutase (PAA), transferred in liquid nitrogen and subsequently stored at -80°C until the Western blot analysis, which was performed as previously described in detail [19]. The following antibodies were used: a monoclonal mouse anti-mouse PCNA antibody (1:2000; Dako, Hamburg, Germany), a polyclonal rabbit anti-mouse Casp-3 antibody (1:300; Cell Signaling Technology, Frankfurt, Germany), a polyclonal rabbit anti-mouse VEGFR-2 antibody (1:300; Cell Signaling Technology), a polyclonal rabbit anti-mouse pAKT(1/2/3) antibody (1:100; Santa Cruz Biotechnology, Heidelberg Germany), a polyclonal rabbit anti-mouse AKT antibody (1:500; Cell Signaling Technology), a monoclonal mouse anti-mouse pERK(1/2) antibody (1:300, Abcam, Cambridge, UK), a polyclonal rabbit anti-mouse ERK(1/2) antibody (1:300, Abcam) and a monoclonal mouse anti-mouse β-actin antibody (1:500; Santa Cruz Biotechnology) followed by the corresponding horse radish peroxidase (HRP)-conjugated secondary antibodies (1:5000; GE Healthcare, Freiburg, Germany).

### Ethics statement

All animal care and experimental procedures were approved by the local governmental animal care committee (Landesamt für Verbraucherschutz, Abteilung C Lebensmittel- und Veterinärwesen, Saarbrücken, Germany; Permit Number: 68/2013) and were conducted in accordance with the European legislation on protection of animals (Guide line 2010/63/EU) and the NIH Guidelines for the Care and Use of Laboratory Animals (http://oacu.od.nih.gov/regs/index.htm. 8th Edition; 2011).

### Aortic ring assay

Geraniol action on vascular sprout formation was analyzed in a rat aortic ring assay [20]. Aortic rings from five male Sprague Dawley rats (250-300g body weight) were embedded in 200μL Matrigel (Basement Membrane Matrix; BD Biosciences) in 48-well plates. After polymerization of the Matrigel at 37°C for 20min, the wells were overlaid with 800μL culture medium containing vehicle (DMSO; control) 100, 200 or 400μM geraniol. The plates were cultured at 37°C for 6 days with medium change on day 3. All assays were done with 8 aortic rings per group. The developing vascular sprouts were visualized by phase-contrast microscopy (Biozero BZ-8000; Keyence) and the area (mm^2^) of the outer aortic vessel sprouting was quantified by means of the software package BZ Analyzer (Biozero, Keyence).

### In vivo analysis of tumor vascularization and growth

The anti-angiogenic action of geraniol on CT26 tumor spheroids was analyzed in the dorsal skinfold chamber model using intravital fluorescence microscopy [19, 21, 22]. Dorsal skinfold chambers were implanted in BALB/c mice with a body weight of 24-27g. The implantation procedure of the chamber has been previously described in detail [23]. After the chamber implantation, the animals could recover from anesthesia and surgical trauma for 48h.

Three days before transplantation, CT26 cell spheroids consisting of 5 x 10^4^ cells were prepared by means of the liquid overlay technique [24, 25]. Before transplantation into the dorsal skinfold chamber, the spheroids were rinsed and stained in PBS supplemented with 2μg/mL Hoechst 33342 for 10min at 37°C.

For the transplantation of the spheroids, we removed the cover glass of the dorsal skinfold chamber and positioned one spheroid onto the striated host muscle within each chamber (n = 16). Thereafter, a group of eight animals was treated daily with 200mg/kg body weight geraniol (dissolved in 100μL corn oil; Sigma-Aldrich) per oral gavage [10]. Eight vehicle-treated animals (100μL corn oil per day) served as controls.

### Intravital fluorescence microscopy

For the analysis of the vascularization of developing CT26 tumors, intravital fluorescence microscopy was performed at days 0, 3, 6, 10, and 14 after spheroid transplantation, as previously described in detail [19, 22]. After the in vivo experiments, the microscopic images were analyzed off-line using the computer-assisted image analysis system CapImage (Dr. Zeintl, Heidelberg, Germany). Quantitative analyses included the measurement of the size (mm^2^) of the tumors and the functional microvessel density, i.e. the length of newly formed red blood cell (RBC)-perfused microvessels per observation area (cm/cm^2^), which contributed to the oxygen supply to the tumors. In addition, the diameter (μm) and the centerline RBC velocity (V_RBC_, μm/s) of these microvessels were measured. V_RBC_ was measured by means of the line-shift-diagram method [26]. For this purpose, a measurement line was drawn in the center of each blood vessel on the monitor screen of the analysis system. Subsequently, the microscopic movie was played for a time period of 10s during which the grey scale value data for each half-frame were read along the measurement lines and transferred in a free image memory in the form of consecutive angled lines. These lines emerged due to the moving erythrocytes and the fluorescently labelled plasma spacing in between. V_RBC_ was then calculated from the slope of these angled lines. Microvascular diameters and V_RBC_ were determined in 20 microvessels within each individual graft. Subsequently, volumetric blood flow (VQ, pL/s) of individual microvessels was calculated from V_RBC_ and diameter (d) for each microvessel as VQ = π * (d/2)^2^ * V_RBC_/K with a Baker-Wayland factor of K = 1.3 [27]. At the end of the 14-day observation period, the animals were sacrificed with an overdose of the anesthetics and tissue samples were harvested for histological and immunohistochemical analyses.

### Histology and immunohistochemistry

Formalin-fixed specimens of the dorsal skinfold chamber preparations were embedded in paraffin. Sections of 1μm thickness were cut and stained with hematoxylin and eosin (HE) according to standard procedures.

Additional sections were stained with a monoclonal rat anti-mouse antibody against the endothelial cell marker CD31 (1:30; Dianova GmbH, Hamburg, Germany), followed by a goat anti-rat IgG Cy3-labeled antibody (1:50; Dianova GmbH). Apoptotic cells within the tumors were detected by a polyclonal rabbit anti-mouse Casp-3 antibody (1:100; Cell Signaling Technology) and proliferating cells by a polyclonal rabbit anti-mouse Ki67 antibody (1:2000; Abcam). Immunoreactive sites were visualized by a biotinylated goat anti-rabbit IgG (ready-to-use; Abcam) followed by Cy3-labeled streptavidin (1:50; Invitrogen, in 10% goat serum). For the simultaneous detection of CD31 and VEGFR-2, sections were stained with a monoclonal rat anti-mouse antibody against CD31 (1:200; Dianova GmbH) and a polyclonal rabbit anti-mouse antibody against VEGFR-2 (1:100; Cell Signaling Technology). A goat anti-rat IgG Alexa Fluor 488-labeled antibody (1:100; Dianova GmbH) and a biotinylated goat anti-rabbit IgG antibody (ready-to-use; Abcam) followed by Cy3-labeled streptavidin (1:50; Invitrogen, in 10% goat serum) served as secondary antibodies. Cell nuclei were stained with Hoechst 33342 (1:500). Subsequently, sections were examined using the Biozero BZ-8000 microscope. Quantitative analyses of the sections included the determination of the microvessel density, i.e. the number of CD31-positive microvessels per observation area (mm^-2^), and the fraction of apoptotic and proliferating cells (%) within the tumors.

### Statistics

Data were tested for normal distribution and equal variance. Differences between two groups were analyzed by the unpaired Student's t-test. Differences between multiple groups were analyzed by ANOVA, which was followed by the Student Newman Keuls test with correction of the alpha error according to Bonferroni probabilities to compensate for multiple comparisons (SigmaStat; Jandel Corporation, San Rafael, CA, USA). All values are expressed as means ± SEM. Statistical significance was accepted for a p-value of P<0.05.

## Results

### Geraniol action on cell viability

**Fig 1:**
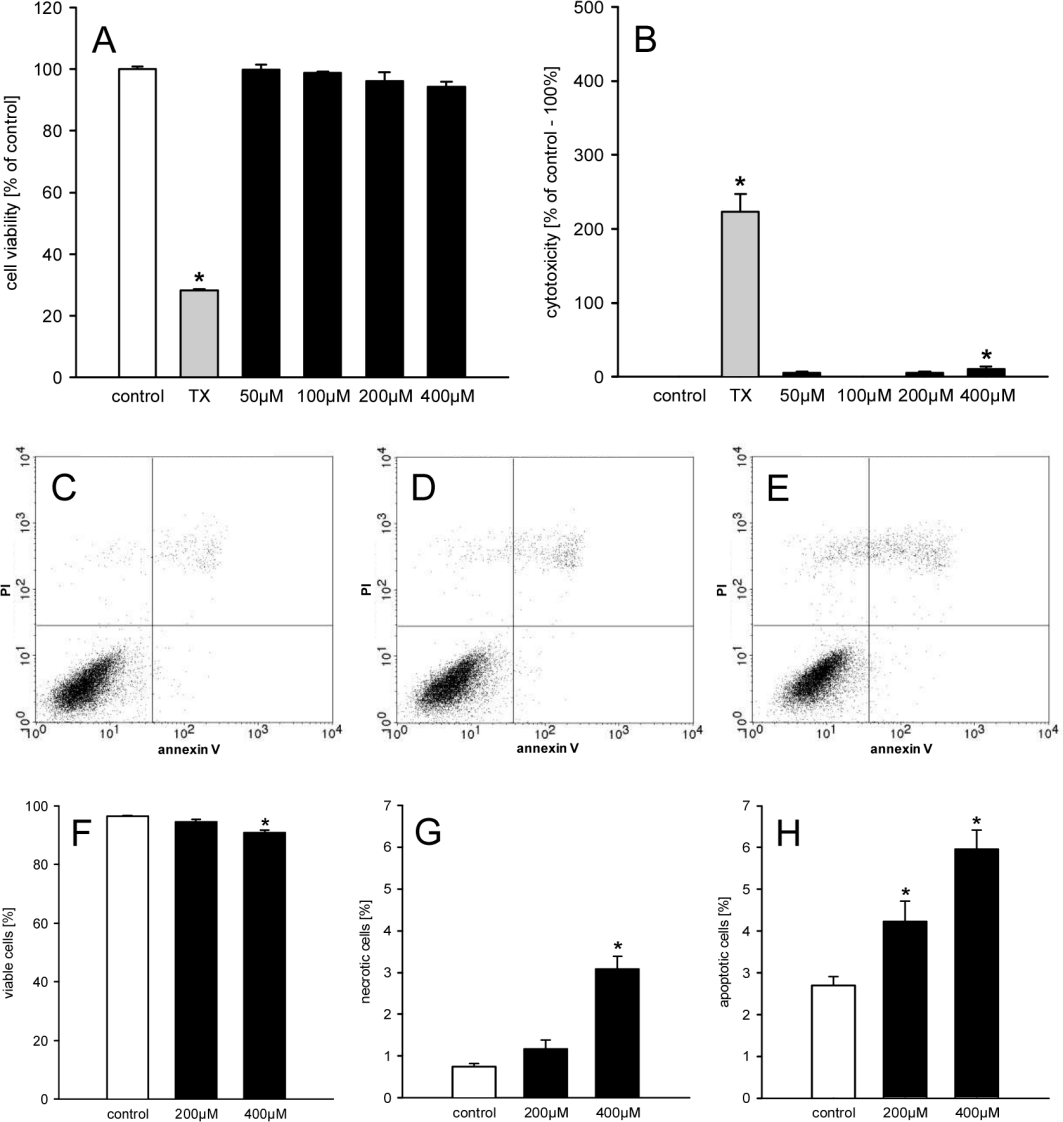
Geraniol action on viability of eEND2 cells. A, B: Cell viability (% of control) (A) and cytotoxicity (% of control– 100%) (B) of eEND2 cells, which were exposed for 24h to different doses (50–400μM; n = 4) of geraniol, Triton X-100 as cytotoxic control (TX) or vehicle (control; n = 4), as assessed by WST-1 assay (A) and LDH release assay (B). Means ± SEM. *P<0.05 vs. control. C-E: Representative graphs from flow cytometry analyses of PI- and annexin V-stained eEND2 cells, which were exposed for 24h to 200μM (D; n = 4) and 400μM (E; n = 4) geraniol or vehicle (control; C; n = 4). F-H: Viable cells (= PI-negative/annexin V-negative; %) (F), necrotic cells (= PI-positive/annexin V-negative; %) and apoptotic cells (PI-negative/annexin V-positive and PI-positive/annexin V-positive; %), as assessed by flow cytometry. Means ± SEM. *P<0.05 vs. control.

In a first step we examined the effect of different doses of geraniol on the viability of eEND2 cells by means of a WST-1 assay. We found that geraniol doses of 50–400μM do not affect the viability of the cells (Fig 1A). Moreover, treatment of the cells in this dose range only showed a significant LDH release into the culture medium at a geraniol concentration of 400μM (Fig 1B). However, the extent of this LDH release (~10%) was rather low. These findings were confirmed by flow cytometric analyses of PI- and annexin V-stained eEND2 cells, which were exposed to 200 or 400μM geraniol (Fig 1C–1H). Once again, they exhibited a high fraction of viable cells (>90%) (Fig 1F). Besides, we found that geraniol dose-dependently induced necrotic and apoptotic cell death in a small fraction of cells (Fig 1G and 1H).

**Fig 2:**
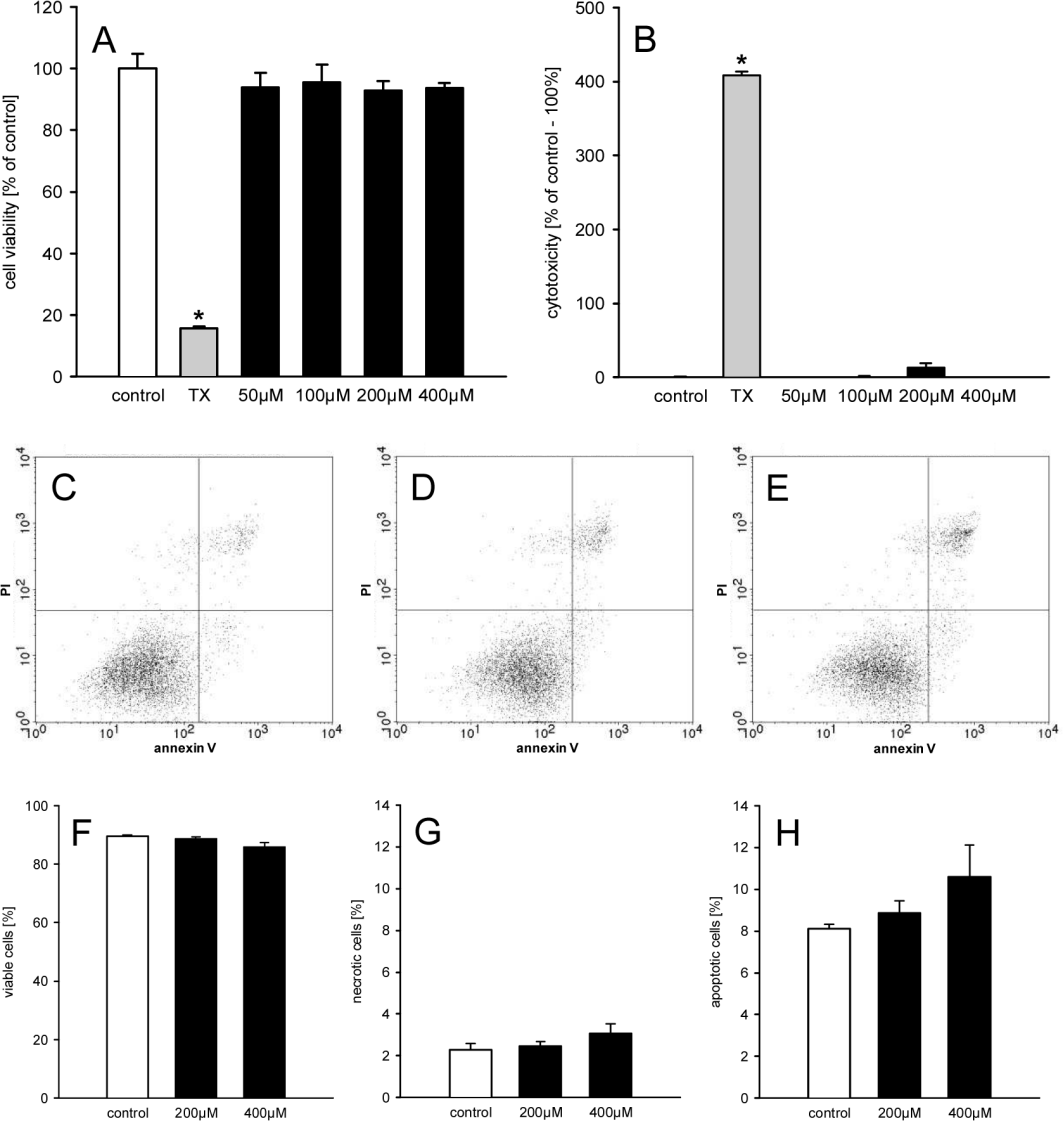
Geraniol action on viability of HDMEC. A, B: Cell viability (% of control) (A) and cytotoxicity (% of control– 100%) (B) of HDMEC, which were exposed for 24h to different doses (50–400μM; n = 4) of geraniol, Triton X-100 as cytotoxic control (TX) or vehicle (control; n = 4), as assessed by WST-1 assay (A) and LDH release assay (B). Means ± SEM. *P<0.05 vs. control. C-E: Representative graphs from flow cytometry analyses of PI- and annexin V-stained HDMEC, which were exposed for 24h to 200μM (D; n = 4) and 400μM (E; n = 4) geraniol or vehicle (control; C; n = 4). F-H: Viable cells (= PI-negative/annexin V-negative; %) (F), necrotic cells (= PI-positive/annexin V-negative; %) and apoptotic cells (PI-negative/annexin V-positive and PI-positive/annexin V-positive; %), as assessed by flow cytometry. Means ± SEM.

In a second set of experiments, we repeated the viability analyses with HDMEC instead of eEND2 cells and made similar observations (Fig 2A–2H). This demonstrates that the results are not specific for murine eEND2 cells, but are also valid for primary endothelial cells of human origin.

### Geraniol action on stress fiber formation and cell migration

**Fig 3:**
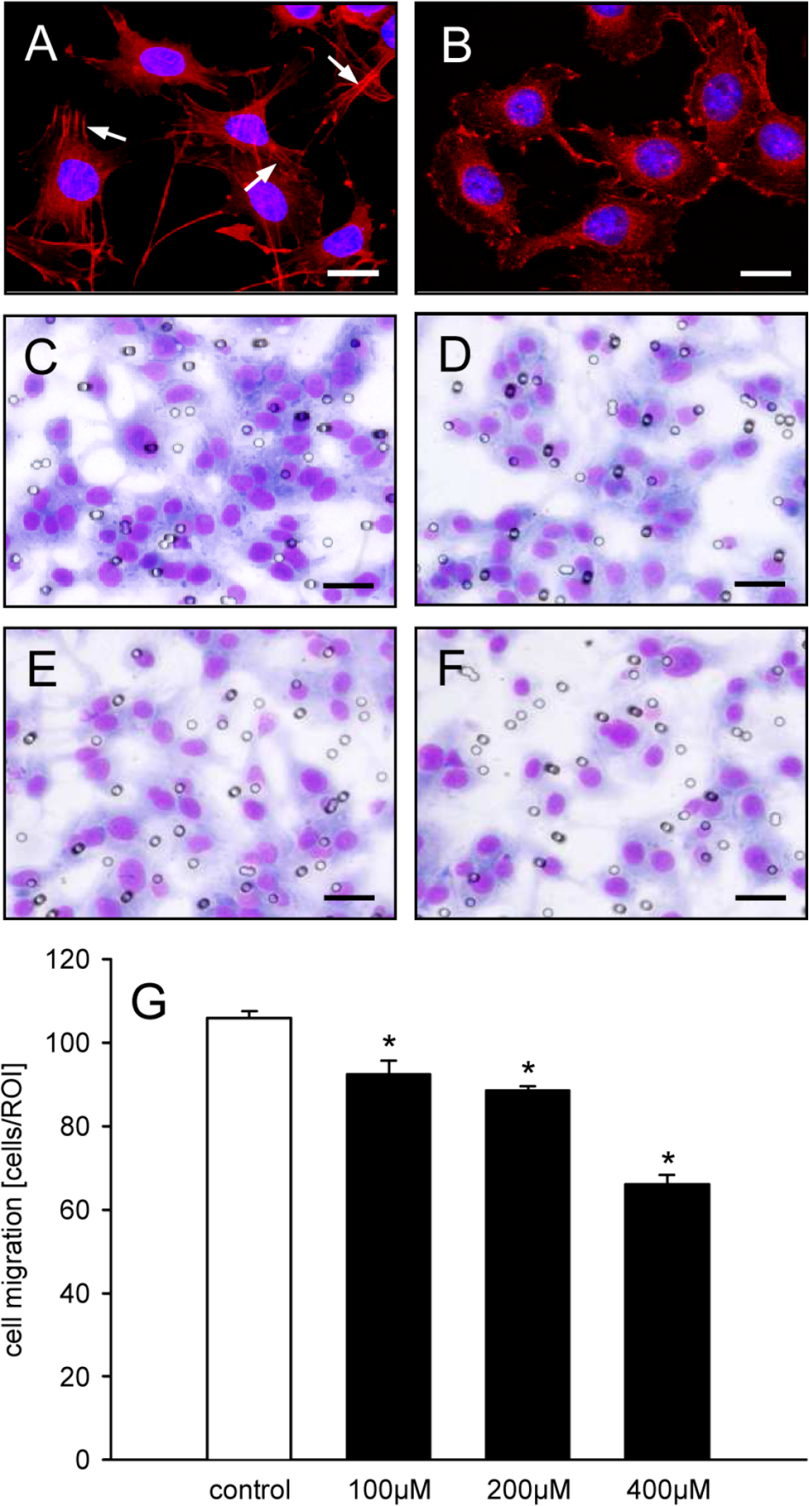
Geraniol action on stress fiber formation and cell migration. A, B: Fluorescence microscopic images of eEND2 cells, which were exposed for 24h to vehicle (A) or 400μM geraniol (B). The cells were stained with Alexa Fluor 568-conjugated phalloidin (red) for the detection of the cytoskeleton. The cell nuclei were stained with Hoechst 33342 (blue). Note that in contrast to the geraniol-treated cells (B) many of the vehicle-treated cells exhibit typical actin stress fibers (A, arrows). Scale bars: 30μm. C-F: Light microscopic images of eEND2 cells, which have migrated through the 8μm pores of the PET filters of the transwell migration assay to the lower membrane surface. The cells were exposed for 24h to vehicle (C), 100μM (D), 200μM (E) or 400μM geraniol (F) and visualized by Diff-Quick staining. Scale bars: 70μm. G: Cell migration (cells/ROI) of eEND2 cells, which were exposed for 24h to different doses (100–400μM; n = 4) of geraniol or vehicle (control; n = 4), as assessed by the transwell migration assay. Means ± SEM. *P<0.05 vs. control.

The analysis of the cytoskeleton of eEND2 cells showed that geraniol treatment reduced the fraction of cells with actin stress fibers (36 ± 3%; n = 4; P<0.05) when compared to vehicle-treated controls (50 ± 3%; n = 4) (Fig 3A and 3B). Moreover, the migration rate of geraniol-treated eEND2 cells was significantly reduced in the transwell migration assay when compared to vehicle-treated controls (Fig 3C–3G).

### Geraniol action on protein expression

**Fig 4:**
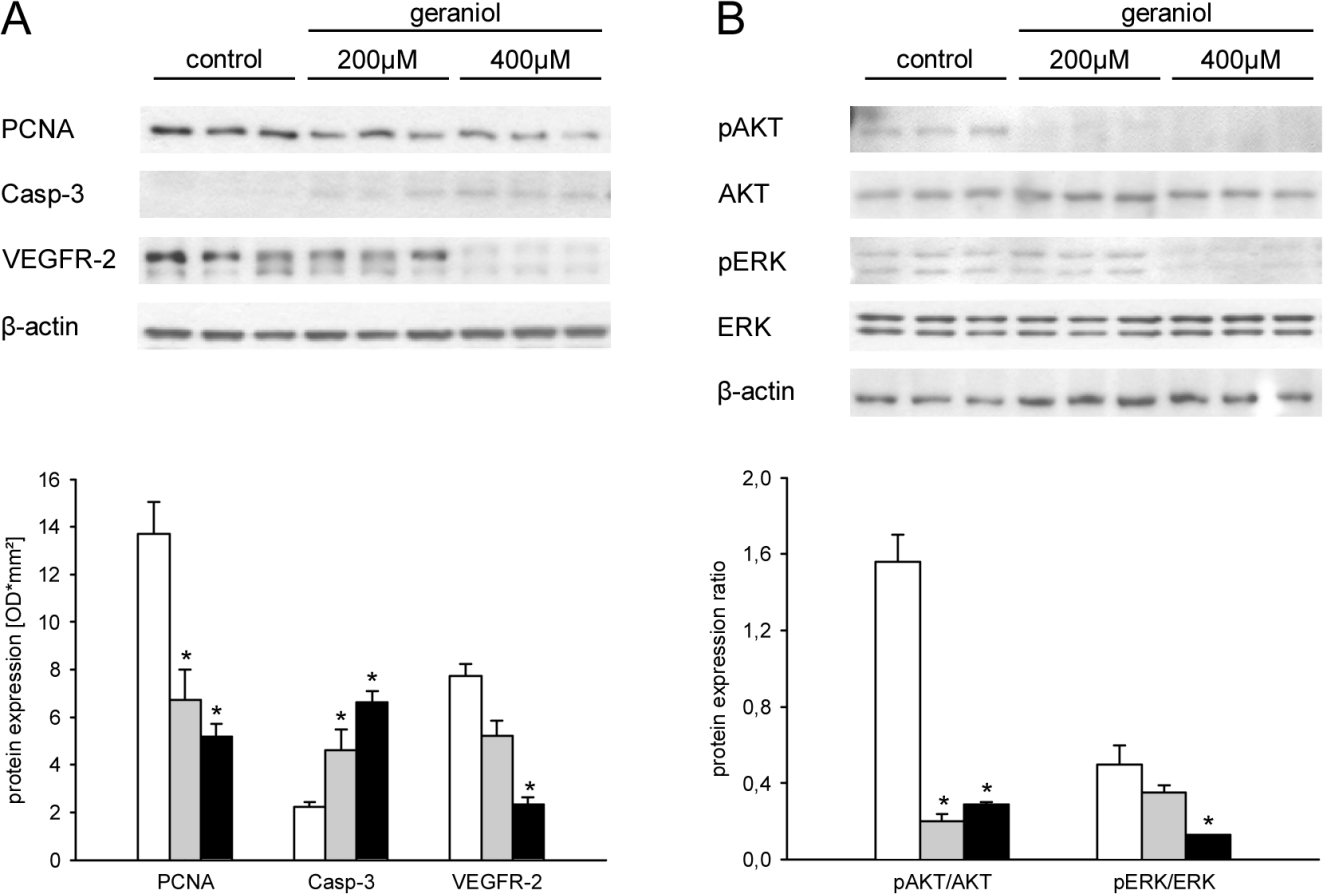
Geraniol action on protein expression. A: Western blot analysis of PCNA, Casp-3 and VEGFR-2 protein expression (optical density (OD)*mm²) of eEND2 cells, which were exposed for 24h to vehicle (white bars; n = 3) or 200μM (grey bars; n = 3) and 400μM geraniol (black bars; n = 3). B: Western blot analysis of pAKT/AKT and pERK/ERK protein expression ratio of eEND2 cells, which were exposed for 24h to vehicle (white bars; n = 3) or 200μM (grey bars; n = 3) and 400μM geraniol (black bars; n = 3). Means ± SEM. *P<0.05 vs. control.

We additionally examined the impact of geraniol treatment on the expression of marker proteins for cell proliferation, angiogenesis and apoptosis by Western blotting. We found that geraniol dose-dependently reduced the expression of PCNA and VEGFR-2 in eEND2 cells when compared to vehicle-treated controls (Fig 4A). In contrast, expression of the cleaved form of caspase-3 was elevated in eEND2 cells, which were exposed to geraniol (Fig 4A). These differences in protein expression were associated with a significantly reduced pAKT/AKT and pERK/ERK ratio (Fig 4B), indicating the suppression of VEGFR-2-induced phospho-regulated signaling pathways in geraniol-treated cells.

### Geraniol action on vascular sprouting

**Fig 5:**
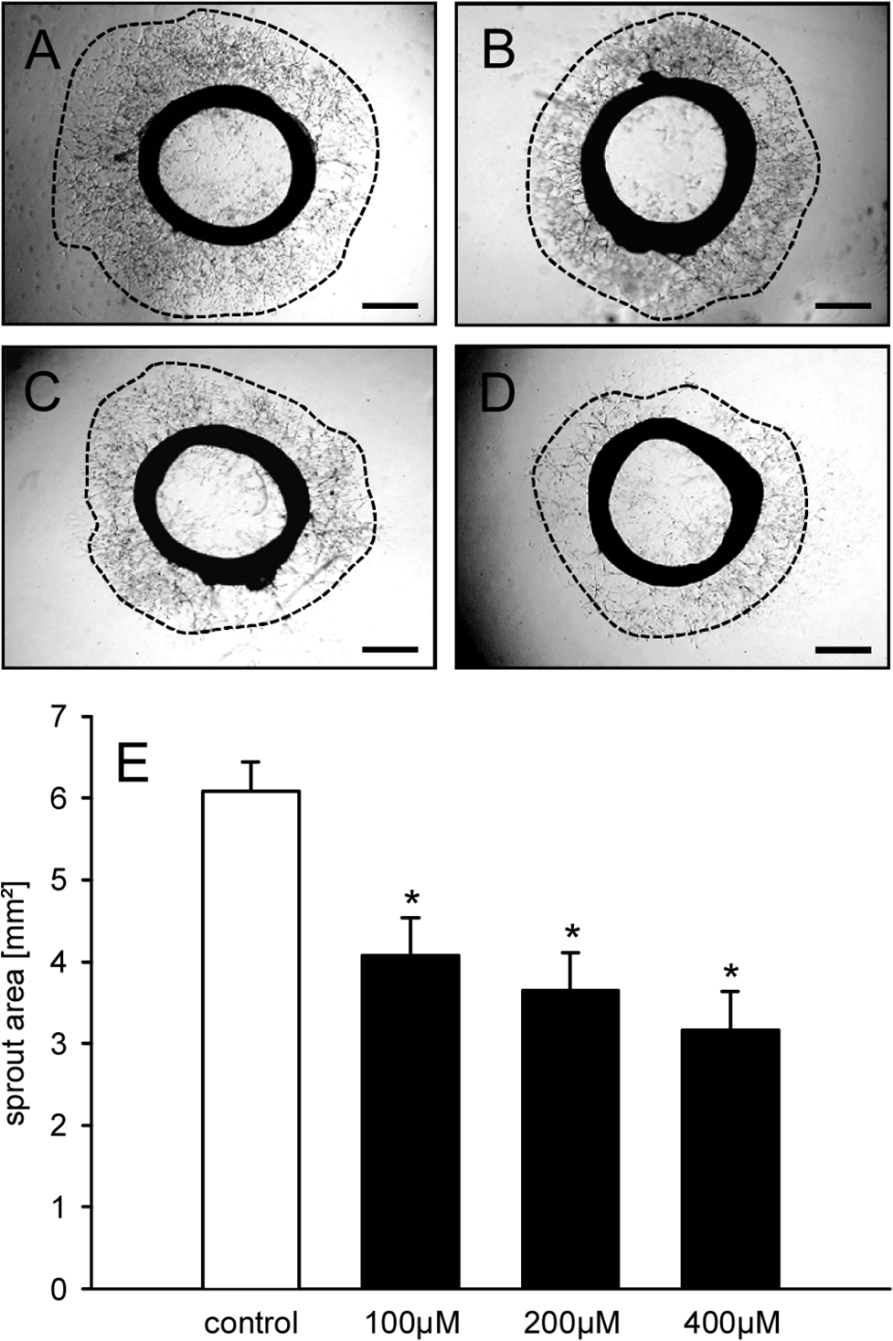
Geraniol action on vascular sprouting. A-D: Phase-contrast microscopic images of rat aortic rings with vascular sprouting (borders marked by broken line) upon 6 days of treatment with vehicle (A), 100μM (B), 200μM (C) or 400μM geraniol (D). Scale bars: 700μm. E: Sprout area (mm^2^) of the outer aortic sprouting, as assessed by phase-contrast microscopy and computer-assisted image analysis. The aortic rings were exposed to vehicle (control; n = 8) or increasing concentrations of geraniol (100–400μM; n = 8) for 6 days. Means ± SEM. *P<0.05 vs. control.

To analyze whether the above described geraniol effects suppress vascular sprout formation, we next performed a rat aortic ring assay. The cultivation of aortic rings in Matrigel induced the growth of vascular sprouts out of the aortic wall, which finally developed a dense network of tubular vessel-like structures (Fig 5A–5D). Of interest, treatment with geraniol effectively inhibited this process. Accordingly, geraniol-treated aortic rings exhibited a significantly reduced vascular sprout area at day 6 of incubation in comparison to vehicle-treated controls (Fig 5E).

### Geraniol action on tumor vascularization and growth

**Fig 6:**
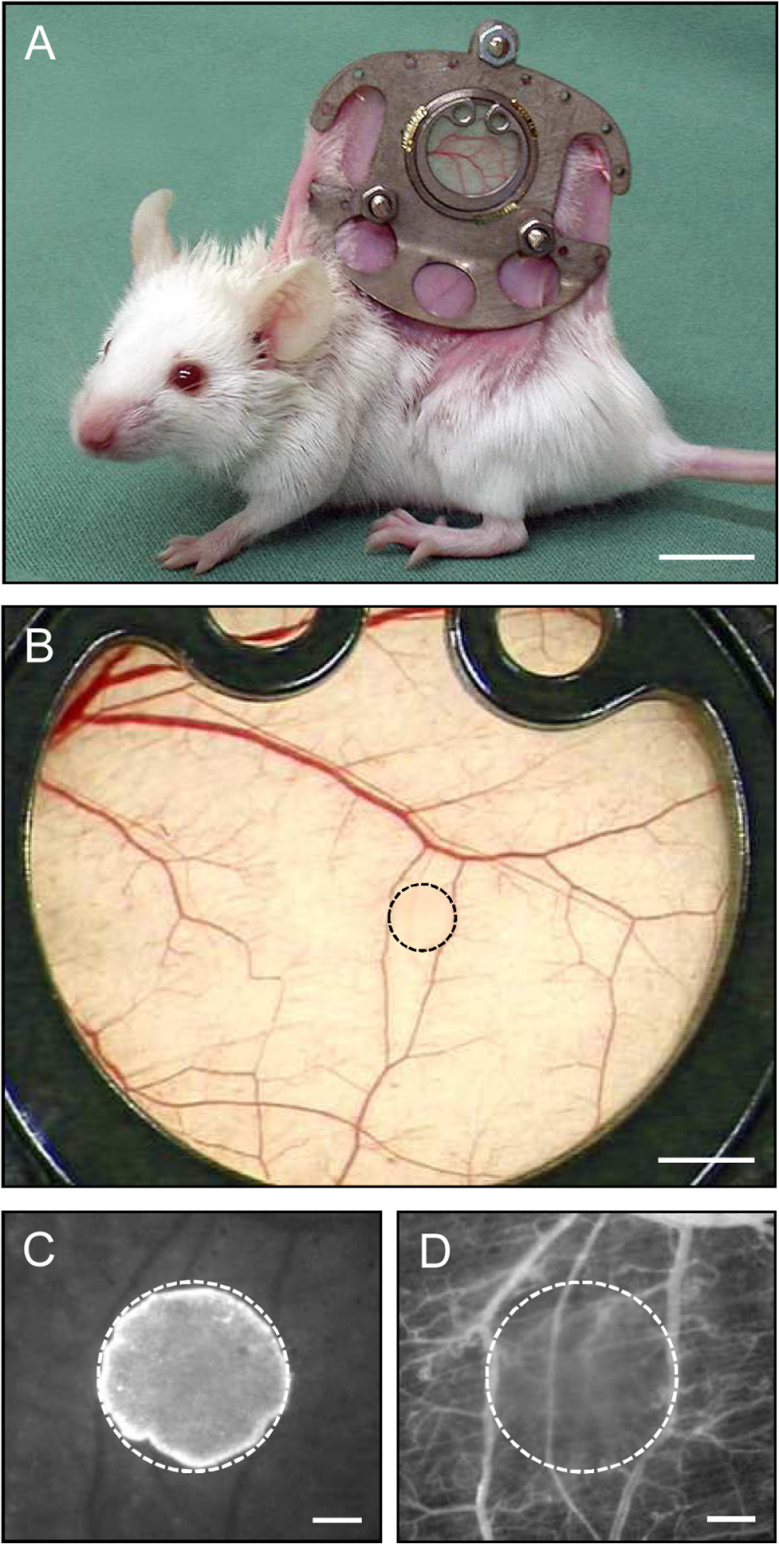
Dorsal skinfold chamber model for the in vivo analysis of tumor angiogenesis. A: BALB/c mouse with a dorsal skinfold chamber (weight: ~2g). B: Observation window of a dorsal skinfold chamber directly after transplantation of a CT26 tumor cell spheroid (border marked by broken line). C, D: Intravital fluorescence microscopy of the tumor cell spheroid (border marked by broken line) in B. Because the cell nuclei of the spheroid were stained with the fluorescent dye Hoechst 33342 before transplantation, the implant can easily be differentiated from the non-stained surrounding host tissue of the chamber using ultraviolet light epi-illumination (C). Blue light epi-illumination of the identical region of interest as in C with contrast enhancement by intravascular staining of plasma with 5% FITC-labeled dextran 150,000 i.v. allows the visualization of the microvasculature surrounding the spheroid (D). Scale bars: A = 10mm; B = 1.4mm; C, D = 250μm.

**Fig 7:**
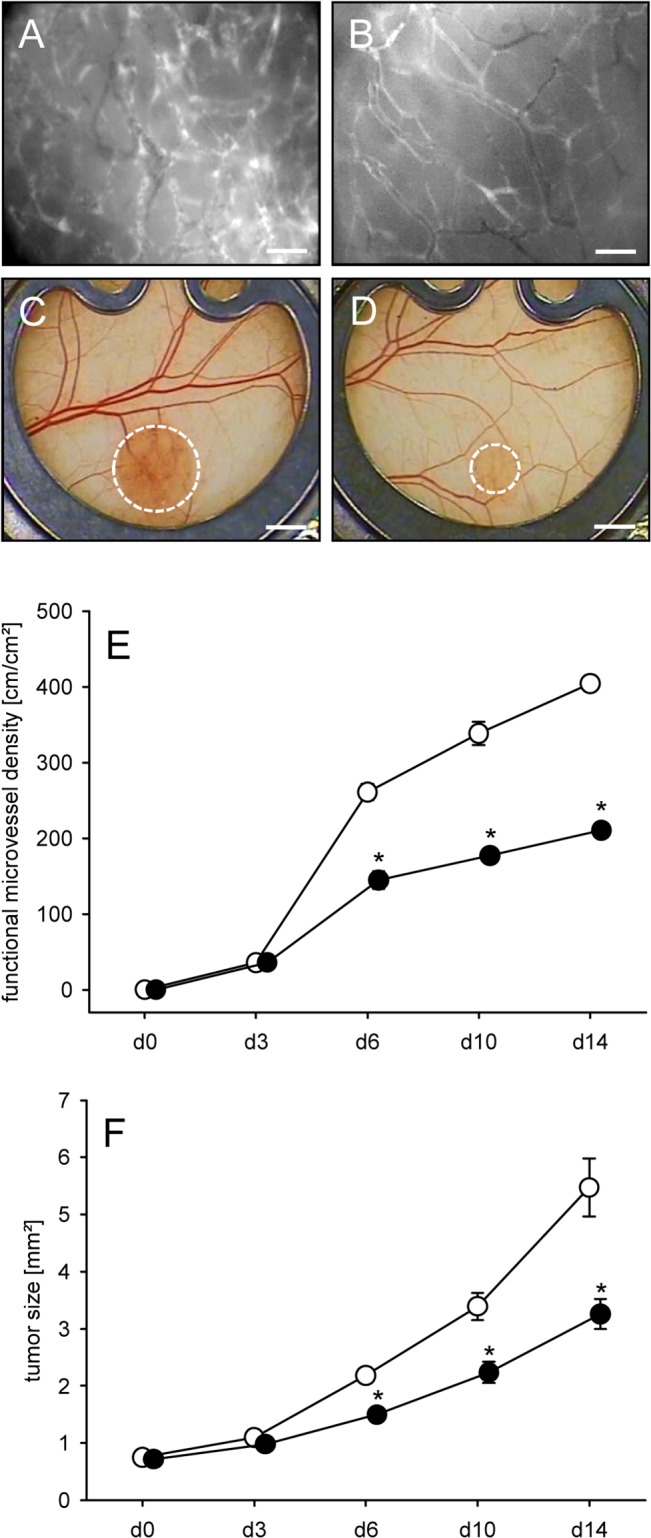
Geraniol action on tumor vascularization and growth. A, B: Intravital fluorescence microscopic images of the newly developed microvascular network within CT26 tumors at day 14 after implantation into the dorsal skinfold chamber of a vehicle-treated control mouse (A) and a geraniol-treated animal (B). Blue light epi-illumination with contrast enhancement by 5% FITC-labeled dextran 150,000 i.v.. Scale bars: 50μm. C, D: Stereo microscopic images of CT26 tumors (borders marked by broken line) at day 14 after transplantation of spheroids into the dorsal skinfold chamber of a vehicle-treated (C) and a geraniol-treated animal (D). Scale bars: 1.4mm. E, F: Functional microvessel density (cm/cm^2^) (E) and size (mm^2^) (F) of CT26 tumors in dorsal skinfold chambers of vehicle-treated (white circles; n = 8) and geraniol-treated BALB/c mice (black circles; n = 8), as assessed by intravital fluorescence microscopy and computer-assisted off-line analysis. Means ± SEM. *P<0.05 vs. control.

The effect of geraniol on the vascularization and growth of CT26 tumor spheroids was analyzed in the dorsal skinfold chamber model (Fig 6A–6D). Directly after transplantation, the tumor spheroids in geraniol-treated and vehicle-treated mice exhibited a homogeneous round shape and a comparable initial size of 0.72 ± 0.05mm^2^ and 0.75 ± 0.07mm^2^, respectively. During the observation period, newly formed microvessels grew into all grafts. However, the process of blood vessel development was markedly suppressed in geraniol-treated animals. In this group, tumor spheroids presented with a significantly lower functional microvessel density between day 3 to day 14 after transplantation when compared to vehicle-treated controls (Fig 7A, 7B and 7E). Furthermore, geraniol-treated tumors exhibited a reduced growth rate over time and, thus, a significantly smaller tumor size of ~3mm² at day 14 when compared to a tumor size of ~5mm² in controls (Fig 7C, 7D and 7F).

More detailed analyses of the newly formed microvascular networks inside the tumor spheroids revealed comparable initial microvessel diameters of 14–15μm in both experimental groups at day 3, which progressively decreased over time (Table 1). Of interest, the microvessels in geraniol-treated tumors exhibited a reduced V_RBC_ (Table 1). Accordingly, calculated values of VQ were also significantly lower for the microvessels of geraniol-treated tumors when compared to vehicle-treated controls (Table 1).

**Table 1:** Microhemodynamic parameters of tumor microvessels. Diameter (μm), V_RBC_ (μm/s) and VQ (pL/s) of newly formed microvessels in CT26 tumors of vehicle-treated (control; n = 8) and geraniol-treated BALB/c mice (geraniol; n = 8), as assessed by intravital fluorescence microscopy and computer-assisted off-line analysis.

	d3	d6	d10	d14
**Diameter [μm]**				
Control	15.3 ± 0.6	12.4 ± 0.2	11.5 ± 0.2	11.4 ± 0.2
Geraniol	14.4 ± 0.5	12.8 ± 0.2	11.8 ± 0.2	11.7 ± 0.2
**V**_**RBC**_ **[μm/s]**				
Control	126.9 ± 6.4	150.5 ± 7.6	166.4 ± 16.7	183.1 ± 20.6
geraniol	118.5 ± 8.4	131.6 ± 9.5	134.5 ± 12.3	133.9 ± 7.7*
**VQ [pL/s]**				
control	18.4 ± 1.6	14.8 ± 0.9	14.1 ± 1.3	15.1 ± 1.5
geraniol	12.0 ± 0.7*	12.3 ± 1.2	10.2 ± 1.1*	10.7 ± 0.9*

Means ± SEM.

*P<0.05 vs control.

**Fig 8:**
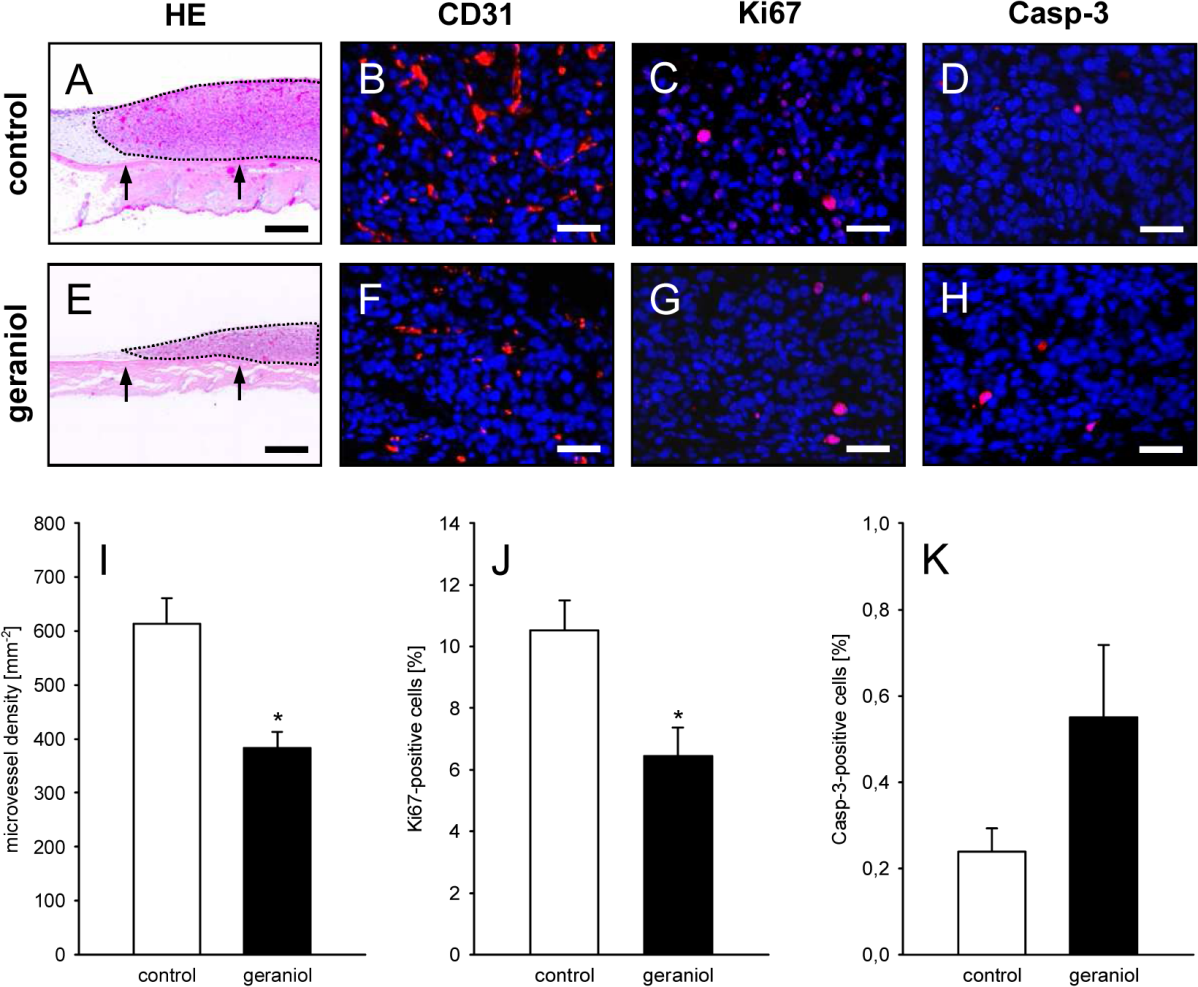
Histological and immunohistochemical analysis of tumors. A, E: HE-stained cross sections of CT26 tumors (borders marked by dotted line) at day 14 after transplantation of tumor spheroids onto the striated muscle tissue (arrows) within the dorsal skinfold chamber of a vehicle-treated control mouse (A) and a geraniol-treated animal (E). Scale bars: 300μm. B, C, D, F, G, H: Immunohistochemical detection of CD31 (B, F, red), Ki67 (C, G, red) and Casp-3 (D, H, red) in CT26 tumors at day 14 after transplantation of tumor spheroids into the dorsal skinfold chamber of a vehicle-treated control mouse (B, C, D) and a geraniol-treated animal (F, G, H). Sections were stained with Hoechst 33342 to identify cell nuclei (blue). Scale bars: 40μm. I-K: Microvessel density (mm^-2^) (I), Ki67-positive cells (%) (J) and Casp-3-positive cells (%) (K) in CT26 tumors in dorsal skinfold chambers of vehicle-treated (white bars; n = 8) and geraniol-treated BALB/c mice (black bars; n = 8), as assessed by quantitative immunohistochemical analysis. Means ± SEM. *P<0.05 vs. control.

**Fig 9:**
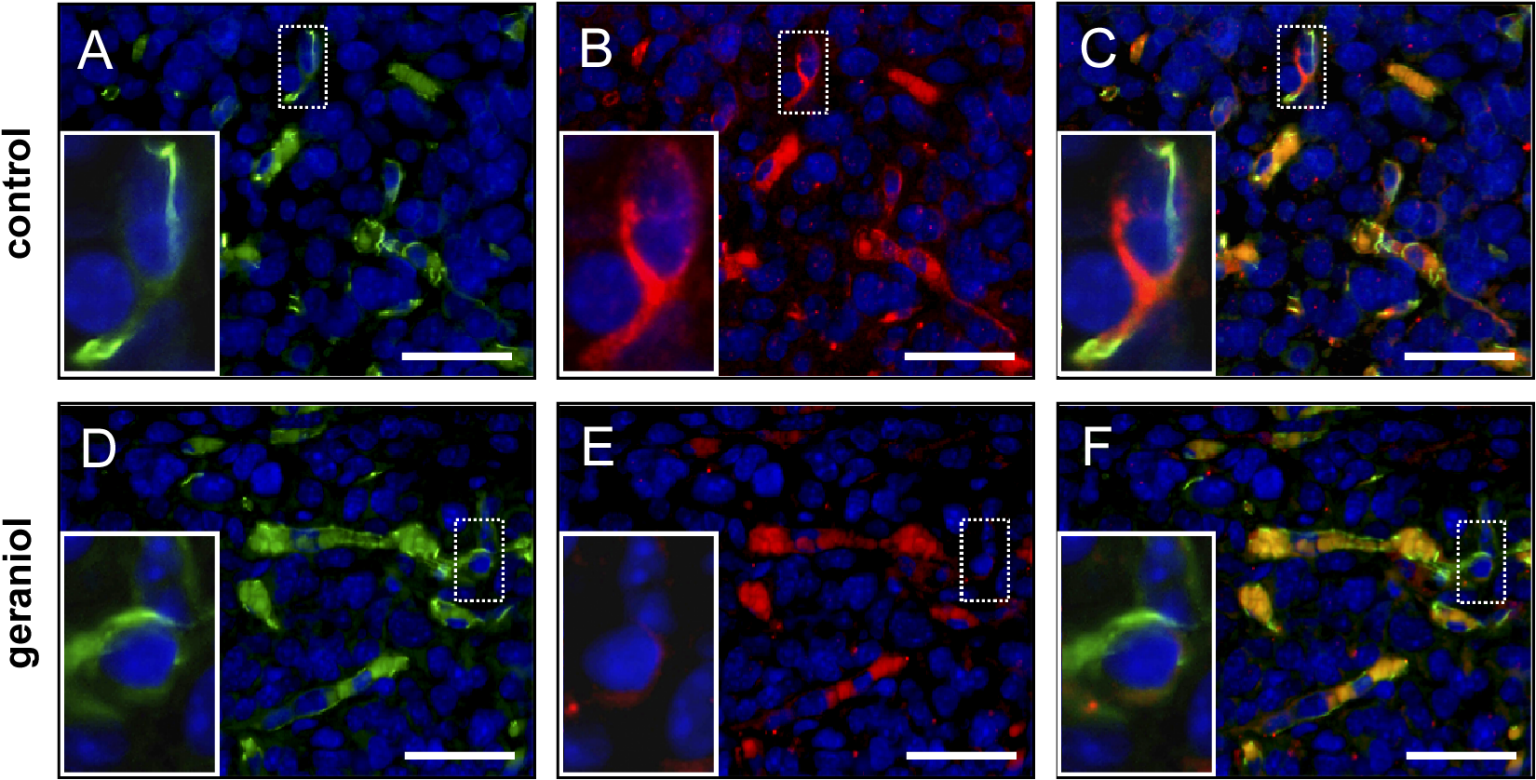
Immunohistochemical analysis of tumor microvessels. Immunohistochemical detection of endothelial CD31 (A, D, green) and VEGFR-2 (B, E, red) of microvessels within CT26 tumors at day 14 after transplantation of tumor spheroids into the dorsal skinfold chamber of a vehicle-treated control mouse (A-C) and a geraniol-treated animal (D-F). Sections were stained with Hoechst 33342 to identify cell nuclei (blue). C and F are merges of A, B and D, E. Erythrocytes in the vessel lumina are unspecifically stained (C, F, orange color). Note that the endothelium of microvessels within the geraniol-treated tumor exhibits a markedly reduced expression of VEGFR-2 (E, insert = higher magnification of dotted ROI) when compared to those within the vehicle-treated control tumor (B, insert = higher magnification of dotted ROI). Scale bars: 35μm.

At day 14 after spheroid transplantation further histological and immunohistochemical analyses of tissue samples from the dorsal skinfold chamber preparations were performed. It was found that geraniol-treated tumor spheroids exhibited a markedly reduced size on HE-stained cross sections when compared to controls (Fig 8A and 8E). Moreover, the density of CD31-positive microvessels (Fig 8B, 8F and 8I) and the number of Ki67-positive proliferating cells (Fig 8C, 8G and 8J) was significantly lower in geraniol-treated spheroids. In both groups, we detected a very low fraction of Casp-3-positive apoptotic cells, which, however, did not differ between geraniol-treated and vehicle-treated control spheroids (Fig 8D, 8H and 8K). More detailed analyses of the tumor microvasculature revealed that geraniol-treated CD31-positive endothelial cells exhibited a reduced expression of VEGFR-2 when compared to vehicle-treated controls (Fig 9A–9F).

## Discussion

The natural compound geraniol is an anti-cancer agent, which suppresses the growth of various tumors, such as prostate cancer [28], colon cancer [29], lung cancer [30], hepatic cancer [31], pancreatic cancer [32] as well as oral and skin cancer [15, 33, 34]. By now, this anti-tumor activity has been mainly attributed to direct geraniol effects on tumor cells. These include primarily the inhibition of cell proliferation and the induction of apoptotic cell death [11, 32]. Herein, we now provide for the first time evidence that geraniol also exerts strong anti-angiogenic activity and, thus, may be beneficial in cancer therapy by targeting the development of new blood vessels inside primary tumors and their metastases.

During the last years, several anti-angiogenic compounds have been clinically approved by the US Food and Drug Administration, such as the humanized monoclonal VEGFR antibody bevacizumab or the tyrosine kinase inhibitor sorafenib [35]. However, they exhibit limited efficacy, because angiogenesis underlies multiple regulatory pathways, which can compensate the inhibition of specific molecular targets. This problem may be overcome by the application of pleiotropic phytochemical agents, which affect different steps of the angiogenic process and additionally exert direct inhibitory effects on tumor cells. Indeed, phytochemicals, such as geraniol, may be attractive candidates for future adjuvant tumor therapy. In fact, their continuous low-dose application may maintain tumor control by targeting excessive pathological angiogenesis without inducing severe side effects [36].

Recently, Vinothkumar et al. [17] could demonstrate that geraniol inhibits the cellular expression of VEGF, which is well known as the essential stimulator of tumor angiogenesis [37]. However, they did not study the effects of geraniol on blood vessel formation. For this purpose, we herein exposed in a first step eEND2 cells to different non-toxic geraniol concentrations. These endothelial-like cells are derived from a murine hemangioma and have been previously used to evaluate the efficiency of anti-angiogenic test compounds [38, 39]. Of interest, we found that geraniol targets multiple angiogenic mechanisms. In fact, geraniol reduced dose-dependently proliferation of eEND2 cells, as indicated by a downregulation of PCNA expression. In addition, geraniol reduced the formation of actin stress fibers in these cells. This may explain its inhibitory action on cell migration, which is crucially dependent on actin filament reorganization [40].

VEGFR-2 is known to mediate the full spectrum of VEGF responses in endothelial cells, including cell survival, proliferation, migration and tube formation [41]. Accordingly, we specifically studied the expression of this receptor by Western blot analyses, which revealed a significant downregulation of VEGFR-2 expression in geraniol-treated eEND2 cells when compared to vehicle-treated controls. In line with this result we further found a marked suppression of the downstream phospho-regulated AKT and ERK signaling pathways in geraniol-treated cells. These findings show that the anti-angiogenic action of geraniol is caused by the suppression of VEGF/VEGFR-2 signaling. Recent studies indicate that this may be mediated by pleiotropic geraniol effects on different intracellular targets. For instance, Galle et al. [30] found that geraniol decreases the cellular level of 3-hydroxy-3-methylglutaryl coenzyme A (HMG-CoA) reductase, which is the rate-limiting enzyme of the mevalonate pathway. On the other hand, geraniol activates peroxisome proliferator-activated receptor (PPAR)-γ [42]. Both mechanisms have been shown to inhibit VEGF-driven angiogenesis under various pathological conditions [43–46].

The results obtained in cell-based angiogenesis assays should always be interpreted with caution, because different endothelial cell lines or primary endothelial cells may markedly differ in terms of their endothelial phenotype [47]. Accordingly, it is mandatory to confirm such results in appropriate control systems. For this purpose, we performed in a next step a rat aortic ring assay. This approach is performed with endothelial cells of freshly isolated aortic rings, which are not pre-selected by passaging and are not in a proliferative state [48]. Moreover, the vessels growing out of the rings exhibit a histomorphology, which is similar to newly formed microvessels in situ, because they also recruit perivascular smooth muscle cells and pericytes [49]. Therefore, the aortic ring assay is considered to mimic closely in vivo angiogenesis. Using this assay, we could show that geraniol inhibits the sprouting activity of microvessels, resulting in a significantly reduced sprout area when compared to controls.

Based on our in vitro results, we finally assessed the action of geraniol on tumor angiogenesis in the dorsal skinfold chamber model of BALB/c mice. The mice were daily treated with oral gavage of geraniol at a dose of 200mg/kg, because this dose has previously been shown to effectively suppress tumor incidence in a rat model of renal carcinogenesis [10]. Moreover, it was well tolerated during long-term treatment over 16 weeks and even induced the downregulation of serum toxicity markers [10]. In line with these findings, we could not detect any changes in the behaviour of the animals when compared to vehicle-treated controls. They exhibited normal feeding, cleaning and sleeping habits. Thus, severe side effects of geraniol treatment can be excluded in the present study. Nonetheless, as any other systemic anti-angiogenic therapy, geraniol treatment may affect physiological angiogenesis in the female reproductive system or during regenerative processes, such as wound healing. Therefore, it will be necessary to clarify the safety profile of this compound in more detail in future toxicity studies.

To assess the vascularization of newly developing CT26 tumors, we used the technique of intravital fluorescence microscopy. This allowed us to analyze the architecture of the tumor microvasculature as well as microhemodynamic parameters. Hence, we could demonstrate that geraniol treatment does not only reduce the functional microvessel density of the tumors, but also markedly affects blood perfusion of individual tumor microvessels. The latter result may be explained by the anti-angiogenic action of geraniol, resulting in less interconnections between newly developing microvessels within the tumors and the supplying and draining blood vessels of the host tissue. This may have increased the resistance and, thus, deteriorated the blood flow conditions within the tumor microvasculature. On the other hand, geraniol may have reduced the metabolic demand of the tumor tissue by direct inhibitory effects on tumor cells. Accordingly, immunohistochemical analyses revealed a lower number of Ki67-positive proliferating cells inside geraniol-treated tumors. In line with our in vitro results we further detected a downregulation of endothelial VEGFR-2 expression in the tumor microvessels. In contrast, geraniol-treated and vehicle-treated tumors exhibited a comparably low fraction of apoptotic cells. These findings indicate that the anti-angiogenic and anti-proliferative action of geraniol, rather than its known pro-apoptotic properties, contributed to the growth inhibition of geraniol-treated tumors in the present setting.

In summary, this study demonstrates for the first time that geraniol is an anti-angiogenic compound, which suppresses endothelial cell proliferation, migration and sprout formation. In addition, geraniol inhibits tumor angiogenesis, which may markedly contribute to its chemopreventive and therapeutic effectiveness in experimental tumor studies. Thus, geraniol should be further tested for its suitability as an anti-angiogenic component of novel treatment regimens in clinical cancer therapy.
